# Supplementary material for: Predictive value of hemogram parameters in malignant transformation of the endometrium in patients with different risk factors
Source: PLoS One. 2023 Jan 10;18(1):e0279224. doi: 10.1371/journal.pone.0279224 (PMC9831310; doi:10.1371/journal.pone.0279224)
Supplement: Attachment — Submitted filename: MR Tomasz Urbanowicz.docx [file pone.0279224.s001.docx]

MR Tomasz Urbanowicz

Academic Editor of PLOS ONE

Subject: Peer review process

Date: October 27, 2022

**Manuscript #:** PONE-D-22-22555

**Title:** Predictive value of hemogram parameters in malignant transformation of the endometrium in patients with different risk factors

**Authors:** Aysun Firat; Aysegul Ercan; Cengiz Mordeniz; Fatma Ferda Verit Atmaca

**Article type:** Research Article

Dear Editor Dr. Urbanowicz,

The present study entitled as ‘Predictive value of hemogram parameters in malignant transformation of the endometrium in patients with different risk factors’ is a clinical research, and the subject of the study is current and also original, since it is on one of the most controversial subjects in inflammation and cancer. The authors investigate neutrophil/ lymphocyte and platelet/lymphocyte ratios (NLR and PLR) in different stages of endometrial pathology in a range from polyps to atypia and further to the carcinoma as a sequence. There is an ongoing debate over this topic, and when I searched at PubMed for your journal’s previously published manuscripts for similar topics (<https://pubmed.ncbi.nlm.nih.gov/?term=PLOS+ONE+NLR+PLR>+); I concluded that the subject is in your scope of interest since there were a total of 75 studies on NLR / PLR in your previous issues, but none of them is about a gynecologic malignancy such as endometrium carcinoma. Therefore, this study worth reading and is appropriate for your readers as well. As for my general PubMed search on NLR and PLR changes in endometrial cancer, there were scarce sources and each with different results, mostly claiming a positive correlation between cancer and NLR and/or PLR. In the present study, authors conclude that their findings support the impact of inflammation on malign transformation from normal endometrial mucosa to atypia and carcinoma. However, NLR and PLR values showed no statistical difference in their patients. Instead, thrombocytosis may have a predictive role in this disease. Different from the small amount of studies previously published in English-written literature; reporting negative/null results on NLR and PLR and suggesting remarkable thrombocytosis from atypia to carcinoma sequence seem valuable and I believe that the present findings will contribute to the existing data available at the recent literature. Therefore, the results of the study is also interesting, original and worth reading. In general, the study is well-designed, volume and the period of study are both impressive (over 400 patients and a 10-year experience). The title reflects the main text body and the abstract. Whole text including the sections, as Abstract, Introduction, Methods, Results and Discussion, are well-designed and written. Ethics’ Committee-approval is stated in the Methods. The results of other studies and the difference of this study is well discussed, and the possible mechanisms in etiopathology are also mentioned. Statistical analyses are well-done, and the tests choosen for comparing groups (M-W U, K-W, ANOVA and Tukey HSD) are all appropriate. Tables are informative and summarizes the data well. The strength and weaknesses of the study are stated in concordance with STROBE guideline criteria. References are proper and up to date, and numbered in the order in square brackets that they appear in the text (concordant with your writing rules); but the writing style should be revised. Furthermore, a revision is still necessary and the points to be clarified are summarized in rows below.

As a conclusion, the study will contribute to current literature discussing the possible mechanisms of dysplasia-cancer sequence, and can be acceptable for publication in your journal since it includes important data and a detailed discussion section on such a debatable subject. In my opinion, it seems to be proper for your readers’ area of interest. It will be my pleasure to re-evaluate this study after a revision.

- Abstract: Please re-write the sentence ‘NLR and PLR values showed no statistical difference in our patients.’ in conclusion section. The last three words seem to be unnecessary.
- Introduction: What do you mean in the last sentence of the last paragraph: ‘An easy, reproducible and simple marker is still needed to estimate the phases of endometrial pathologic lesions, and distinguish them from pathologically normal results’. Pathologically normal? Please re-write or correct.
- Introduction: There is no need for a subtitle in the last paragraph. Delete it.
- Methods: You need to extend the exclusion criteria. You excluded just patients with other inflammatory diseases? Be clear in defining this section.
- Methods: In the second paragraph, you state that high BMI is a risk factor, but you list normal BMI after parenthesis. Please correct.
- Methods: The abbreviation of SD should be written openly first. In the same paragraph ‘…Group comparisons… group or groups?’. Please verify.
- Results: ‘…(Group II) (53.4 years vs 42.2 and 42 years; each, p<0.05)…’ and or vs? In the same section, ‘The groups were compared by calculated NLR and PLR results, and there were no statistically meaningful relation…’ were or was?
- Discussion: In the first paragraph, ‘…menstruation, PCOS, HRT and tamoxifen use etc., and all can be viewed as factors increasing exposure of the endometrium to inflammation [2-4].’ Viewed? What you mean?
- Discussion: In the third paragraph, ‘…Haruma et al. reported this association for only PLR value [23]. An another recent study determined that…’ An another? What you mean?
- References: Please be compatible with the journal’s reference rules. PLOS uses the numbered citation (citation-sequence) method and first six authors, et al. Example: Hou WR, Hou YL, Wu GF, Song Y, Su XL, Sun B, et al. cDNA, genomic sequence cloning and overexpression of ribosomal protein gene L9 (rpL9) of the giant panda (Ailuropoda melanoleuca). Genet Mol Res. 2011;10: 1576-1588. Devaraju P, Gulati R, Antony PT, Mithun CB, Negi VS. Susceptibility to SLE in South Indian Tamils may be influenced by genetic selection pressure on TLR2 and TLR9 genes. Mol Immunol. 2014 Nov 22. pii: S0161-5890(14)00313-7. doi: 10.1016/j.molimm.2014.11.005. A DOI number for the full-text article is acceptable as an alternative to or in addition to traditional volume and page numbers. When providing a DOI, adhere to the format in the example above with both the label and full DOI included at the end of the reference (doi: 10.1016/j.molimm.2014.11.005). Do not provide a shortened DOI or the URL.
- Authors’ contrubution to the study should be personalized such as [Sho Matsubara](https://pubmed.ncbi.nlm.nih.gov/?term=Matsubara%20S%5BAuthor%5D), Data curation, Formal analysis, Writing – original draft, [Seiji Mabuchi](https://pubmed.ncbi.nlm.nih.gov/?term=Mabuchi%20S%5BAuthor%5D), Conceptualization, Data curation, Formal analysis, Writing – original draft,^*^ [Yoshinori Takeda](https://pubmed.ncbi.nlm.nih.gov/?term=Takeda%20Y%5BAuthor%5D), Data curation, Formal analysis, [Naoki Kawahara](https://pubmed.ncbi.nlm.nih.gov/?term=Kawahara%20N%5BAuthor%5D), Data curation, Formal analysis, and [Hiroshi Kobayashi](https://pubmed.ncbi.nlm.nih.gov/?term=Kobayashi%20H%5BAuthor%5D), Supervision

XXXXXXX, Editor

Regards.

Assoc. Prof. Dr. Sema YUKSEKDAG, M.D., PhD

Department of Surgical Oncology at Umraniye Training and Research Hospital; Elmalıkent Mahallesi Adem Yavuz Cad. No:1 Umraniye / Istanbul 34760

Central         : +90 (216) 632 18 18 ext. 1803/ GSM +90 (505) 930 88 25                     
Fax             : +90 (216) 632 71 24 / +90 (216) 632 71 21
Official mail : [isteah24.hh@saglik.gov.tr](mailto:isteah24.hh@saglik.gov.tr) / [drsemayuksekdag.1973@gmail.com](mailto:drsemayuksekdag.1973@gmail.com)

<https://orcid.org/my-orcid?orcid=0000-0001-8646-7047>

<https://scholar.google.com/citations?hl=tr&user=RIOzk_kAAAAJ>

**To Authors**: The present study entitled as ‘Predictive value of hemogram parameters in malignant transformation of the endometrium in patients with different risk factors’ is a clinical research on one of the most controversial subjects in inflammation and endometrial carcinoma. The authors investigate neutrophil/ lymphocyte and platelet/lymphocyte ratios (NLR and PLR) in different stages of endometrial pathology in a range from polyps to atypia and further to the carcinoma sequence. On the contrary to several previous publications on endometrial cancer, you conclude that NLR and PLR values showed no statistical difference in your patients. However, your results still support the impact of inflammation on malign transformation from normal endometrial mucosa to atypia and carcinoma. The subject is of the last 10 years’ debatable topic among clinicians, since the recent publications from different areaa of expertises not always overlap with each other, and there is a scarce published study on endometrial carcinoma sequence. In general, the text is well-designed but there are some points listed below for revision;

- Abstract: Please re-write the sentence ‘NLR and PLR values showed no statistical difference in our patients.’ In conclusion section. The last three words seem to be unnecessary.
- Introduction: What do you mean in the last sentence of the last paragraph: ‘An easy, reproducible and simple marker is still needed to estimate the phases of endometrial pathologic lesions, and distinguish them from pathologically normal results’. Pathologically normal? Please re-write or correct.
- Introduction: There is no need for a subtitle in the last paragraph. Delete it.
- Methods: You need to extend the exclusion criteria. You excluded just patients with other inflammatory diseases? Be clear in defining this section.
- Methods: In the second paragraph, you state that high BMI is a risk factor, but you list normal BMI after parenthesis. Please correct.
- Methods: The abbreviation of SD should be written openly first. In the same paragraphy ‘…Group comparisons… group or groups?’. Please verify.
- Results: ‘…(Group II) (53.4 years vs 42.2 and 42 years; each, p<0.05)…’ and or vs? In the same section ‘The groups were compared by calculated NLR and PLR results, and there were no statistically meaningful relation…’ were or was?
- Discussion: In the first paragraph, ‘…menstruation, PCOS, HRT and tamoxifen use etc., and all can be viewed as factors increasing exposure of the endometrium to inflammation [2-4].’ Viewed? What you mean?
- Discussion: In the third paragraph, ‘…Haruma et al. reported this association for only PLR value [23]. An another recent study determined that…’ An another? What you mean?
- References: Please be compatible with the journal’s reference rules. PLOS uses the numbered citation (citation-sequence) method and first six authors, et al. Example: Hou WR, Hou YL, Wu GF, Song Y, Su XL, Sun B, et al. cDNA, genomic sequence cloning and overexpression of ribosomal protein gene L9 (rpL9) of the giant panda (Ailuropoda melanoleuca). Genet Mol Res. 2011;10: 1576-1588. Devaraju P, Gulati R, Antony PT, Mithun CB, Negi VS. Susceptibility to SLE in South Indian Tamils may be influenced by genetic selection pressure on TLR2 and TLR9 genes. Mol Immunol. 2014 Nov 22. pii: S0161-5890(14)00313-7. doi: 10.1016/j.molimm.2014.11.005. A DOI number for the full-text article is acceptable as an alternative to or in addition to traditional volume and page numbers. When providing a DOI, adhere to the format in the example above with both the label and full DOI included at the end of the reference (doi: 10.1016/j.molimm.2014.11.005). Do not provide a shortened DOI or the URL.
- Authors’ contrubution the study should be personalized such as [Sho Matsubara](https://pubmed.ncbi.nlm.nih.gov/?term=Matsubara%20S%5BAuthor%5D), Data curation, Formal analysis, Writing – original draft, [Seiji Mabuchi](https://pubmed.ncbi.nlm.nih.gov/?term=Mabuchi%20S%5BAuthor%5D), Conceptualization, Data curation, Formal analysis, Writing – original draft,^*^ [Yoshinori Takeda](https://pubmed.ncbi.nlm.nih.gov/?term=Takeda%20Y%5BAuthor%5D), Data curation, Formal analysis, [Naoki Kawahara](https://pubmed.ncbi.nlm.nih.gov/?term=Kawahara%20N%5BAuthor%5D), Data curation, Formal analysis, and [Hiroshi Kobayashi](https://pubmed.ncbi.nlm.nih.gov/?term=Kobayashi%20H%5BAuthor%5D), Supervision

XXXXXXX, Editor
